# Expression of inhibitory receptors and polyfunctional responses of T cells are linked to the risk of congenital transmission of *T*. *cruzi*

**DOI:** 10.1371/journal.pntd.0005627

**Published:** 2017-06-09

**Authors:** Adriana Egui, Paola Lasso, María Carmen Thomas, Bartolomé Carrilero, John Mario González, Adriana Cuéllar, Manuel Segovia, Concepción Judith Puerta, Manuel Carlos López

**Affiliations:** 1 Instituto de Parasitología y Biomedicina López Neyra, Consejo Superior de Investigaciones Científicas (IPBLN-CSIC), Granada, Spain; 2 Laboratorio de Parasitología Molecular, Pontificia Universidad Javeriana, Bogotá, Colombia; 3 Grupo de Inmunobiología y Biología Celular, Facultad de Ciencias, Pontificia Universidad Javeriana, Bogotá, Colombia; 4 Unidad Regional de Medicina Tropical, Hospital Virgen de la Arrixaca, Murcia, Spain; 5 Grupo de Ciencias Básicas Médicas, Facultad de Medicina, Universidad de los Andes, Bogotá, Colombia; FIOCRUZ - Minas, BRAZIL

## Abstract

Congenital *T*. *cruzi* infections involve multiple factors in which complex interactions between the parasite and the immune system of pregnant women play important roles. In this study, we used an experimental murine model of chronic infection with *T*. *cruzi* to evaluate the changes in the expression of inhibitory receptors and the polyfunctionality of T cells during gestation and their association with congenital transmission rate of *T*. *cruzi* infection. The results showed that pregnant naïve mice had a higher percentage of CD4^+^ and CD8^+^ T cells that expressed inhibitory receptors than cells from non-pregnant naïve mice. However, in mice chronically infected with *T*. *cruzi*, gestation induced a significant decrease in the frequency of T cells that expressed or co-expressed inhibitory receptors, as well as an increase in the frequency of polyfunctional CD4^+^ and CD8^+^ T cells. This different behavior may be due to the breakdown in the infected mice of the gestation-induced immune homeostasis, probably to control the parasite load. Remarkably, it was observed that the mothers that transmitted the parasite had a higher frequency of T cells that expressed and co-expressed inhibitory receptors as well as a lower frequency of polyfunctional parasite-specific T cells than those that did not transmit it, even though the parasitemia load was similar in both groups. All together these data suggest that the maternal immune profile of the CD4^+^ and CD8^+^ T cells could be a determining factor in the congenital transmission of *T*. *cruzi*.

## Author summary

Chagas disease or American trypanosomiasis is a complex parasitic disease caused by the protozoan *Trypanosoma cruzi*. This disease that affects approximately 10 million people worldwide may be mother-to-child transmitted which is an important public health problem with great relevance in endemic and non-endemic areas and regions where the vector transmission has been controlled. During gestation, the maternal immune system must defend both the mother and the fetus from infections, while, at the same time, it must tolerate a semiallogenic fetus. This immune homeostasis is characterized by a natural process of immunosuppression that in *T*. *cruzi-*infected pregnant women can lead to an increase in the mother´s parasite load that favors the risk of congenital transmission. In this study, it was determined the immunological modifications induced by gestation in an experimental model of *T*. *cruzi* chronic infection and their influence in the parasite congenital transmission. The results indicate that a *T*. *cruzi* infection induces a reversion of the pregnancy-associated homeostasis. Furthermore, it is shown that females who are not able to reverse the biological profile induced by pregnancy are those that transmit the parasite.

## Introduction

Chagas disease, which is caused by the protozoan *Trypanosoma cruzi*, is a tropical parasitic disease that affects approximately 10 million people worldwide. The disease is primarily transmitted by contact with the infected feces of triatomine bugs [1]. This parasite can also be transmitted by non-vector mechanisms, including blood transfusions, organ transplants, congenital infections, and oral transmission through food contaminated with insect feces [1, 2]. In a *T*. *cruzi* infection, as in other chronic infectious diseases, it has been shown that antigen persistence is associated with a process of a gradual dysfunction of CD8^+^ T cells [3]. The development of the dysfunction in the T cell response is linked to the constitutive expression of several inhibitory receptors that might negatively regulate the function of antigen-specific T cells, thus compromising pathogen control [4, 5]. In Chagas’ disease patients with increased heart involvement, an active silencing of the CD8^+^ T cell response is characterized by an impaired ability to simultaneously produce multiple cytokines (polyfunctionality) and an increase in the frequency of CD8^+^ T cells that co-express inhibitory receptors [3].

Vertical transmission of *T*. *cruzi* is a worldwide problem with great relevance in endemic and non-endemic areas [6]. In this regard, some authors consider the congenital *T*. *cruzi* infection to be an ecological model that involves multiple and complex interactions between the parasite and (i) the immune system of pregnant women in whom the responses depend on genetic and environmental factors, (ii) the placenta, which possesses its own defense mechanisms, and (iii) the immune system of the fetus [7]. In fact, in a case of twins congenitally infected with *T*. *cruzi*, it was reported that a lower level of pro-inflammatory cytokine production was associated with a poorer clinical prognosis in one of the brothers [8].

During gestation, the maternal immune system must defend both the mother and the fetus from infections, while, at the same time, it must tolerate a semiallogenic fetus [9]. This immune homeostasis is mediated by cellular and molecular mechanisms that include the immunosuppressive effects of regulatory T cells and the immunoregulatory capacity of molecules that are involved in the inhibition of specific immune responses [9, 10]. This natural process of immunosuppression in pregnant women infected with *T*. *cruzi* can lead to an increase in the mother´s parasite load, especially during the third trimester of pregnancy, and this process increases the risk of congenital transmission during this time [11, 12]. Taking into consideration that the pregnancy-associated homeostasis can increase the parasitemia and that the lack of a polyfunctional response correlates with a high expression of inhibitory receptors and, in turn, with the progression of the Chagas disease [3], we considered important to assess the immunological changes regarding the polyfunctionality of T cells and the expression of inhibitory receptors during gestation in a murine experimental model of *T*. *cruzi* chronic infection. The obtained results show that the balance between the expression of the inhibitory receptors and the functionality of CD4^+^ and CD8^+^ T cells plays a decisive role in the process of congenital transmission.

## Methods

### Ethics statement

The Ethic Committees from the Instituto de Parasitología y Biomedicina López Neyra (IPBLN) and from Consejo Superior de Investigaciones Científicas (CSIC) reviewed and approved the animal care and protocols used in this study (registry number MCL.2/14) as well as the project entitled “Immunological and molecular approaches for the Chagas’ disease control” (identification number CEEA/2014/MCL/1, n° 13-03-14-48). The experiments were performed in the Animal Experimentation Unit from IPBLN (registry number ES-180210000022) and conducted according to the Ethics Guidelines of the Animal Care Unit Committee of IPBLN-CSIC provided by the European Union Directive 2010/63 and Spanish Legislative Decree 53/2013.

### Mice

Young (6–10 weeks old) female BALB/c mice were purchased from Charles Rivers Laboratories International, Inc. (Paris, France) and housed at the animal facilities of the Institute of Parasitology and Biomedicine “López-Neyra” (Granada, Spain). The mice were maintained in polyethylene cages with food and water *ad libitum*, on a 12 h light/dark cycle at 20–22°C and 40–60% humidity. The animals were divided into the following four groups: uninfected non-pregnant (*n* = 10), uninfected pregnant (*n* = 11), infected non-pregnant (*n* = 11) and infected pregnant (*n* = 22).

### Parasites and experimental infection

The infections were performed by intraperitoneal (i.p.) inoculation with 10^4^ trypomastigotes of DA (MHOM/CO/01/DA; DTU I) or SOL (MHOM/ES/2008/SOL; DTU V) strains of *T*. *cruzi* in 0.1 ml of sterile phosphate-buffered saline (PBS). DA and SOL strains were, respectively, obtained from an infected human donor in the town of Sutatenza, Boyacá, Colombia [13] and an infant infected by congenital transmission in Spain, Infant 1 referred in [14]. Before infection, the parasites were grown and purified from the monolayers of LLC-MK2 monkey kidney fibroblast line, provided by Dra. N. Andrews (New York University) [15]. The control mice received the same volume of sterile PBS.

### Mating and gestation

Healthy (uninfected) mice, 140 and 240 days old, and infected mice in the chronic phase of Chagas disease (70 and 170 days post-infection) were mated. All mice presented *T*. *cruzi-*specific IgG antibodies two months post-infection (S1 Table) measured using an enzyme-linked immunosorbent assay (ELISA) as previously described [16]. The mating was permitted for 5 days by placing one uninfected male and two females in the same cage. The gravid females were euthanized on day 18^th^ of gestation using carbon dioxide inhalation. Blood was collected by cardiac puncture. The fetuses were extracted by caesarean section, washed and stored at -80°C until DNA extraction. The spleens were aseptically removed after dissection and maintained in RPMI-1640 culture medium (Gibco, Grand Island, NY) supplemented with 10% FBS (Gibco), 100 U/ml penicillin, and 10 μg/ml streptomycin (Gibco) until processing.

### Conventional and quantitative PCR strategies for parasite detection

Genomic DNA (gDNA) extraction from whole blood for conventional PCR and qPCR was carried out using Chelex-100 resin (Bio-Rad Laboratories, Hercules, CA) [17]. The DNA extraction from 250 mg of the fetuses’ tissues from the abdominal-thoracic area was performed using a commercial kit for tissue DNA purification (Qiagen, Valencia, CA) according to the manufacturer´s instructions. For parasite detection, conventional PCR was carried out employing gDNA from whole blood from the mothers and from fetuses tissues using the primers S35 (5’-AAATAATGTACGGG(T/G)GAGATGCATGA-3’) and 122 (5’-GGTTCGATTGGGGTTGGTGTAATATA-3’), which are based on the conserved regions of minicircles from *T*. *cruzi* kinetoplast DNA [18, 19], and the primers TcZ1 (5´-CGAGCTCTTGCCCACACGGGTGCT-3´) and TcZ2 (5´-CCTCCAAGCAGCGGATAGTTCAGG-3´), which are based on the satellite DNA of *T*. *cruzi* [20]. The quantitative PCR performed for parasite load detection was made with gDNA extracted from peripheral blood from the infected mothers as template and the previously mentioned TcZ1 and TcZ2 primers. The parasite load was estimated through extrapolation of the values obtained in a standard curve generated using DNA purified from the blood of non-infected mice reconstituted with serial dilutions of *T*. *cruzi* DNA (DA and SOL strains) ranging from 1 to 0.0001 ng.

### Isolation of *T*. *cruzi* soluble antigens (S*Tc*A)

Soluble total protein extract (S*Tc*A) from amastigote/trypomastigote forms of *T*. *cruzi* was obtained as previously described [21]. Briefly, semiconfluent monolayers of monkey kidney fibroblast cells (LLC-MK2) were infected with tripomastigotes form of the Y strain of *T*. *cruzi* (MHOM/BR/1950/Y human isolate) at parasite:cell ratio 4:1 for 12 hours. At 96-120h post-infection, a mixture of amastigotes and trypomastigotes forms were recovered from infected-culture supernatants and washed in PBS. Subsequently, the parasites were resuspended in lysis buffer (50 mM Tris-HCl at pH 7.4, 0.05% Nonidet P-40, 50 mM NaCl, 1 mM phenylmethylsulfonyl fluoride (PMSF), 1 μg/mL leupeptin) and sonicated. Soluble protein extracts were obtained after centrifugation at 10,000 rpm for 20 min at 4°C.

### Flow cytometry and intracellular cytokine staining assays

The following Abs were used for cell surface staining: anti-CD3 PerCP-Cy5.5 (clone 17A2), anti-CD8a allophycocyanin-H7 (clone 53–6.7), anti-CD4 Alexa Flour 700 (clone GK1.5), anti-PD-1 allophycocyanin (clone J43), anti-2B4 FITC (clone 2B4), and anti-CD160 PE-CF594 (clone CNX46-3) (BD Biosciences, San Jose, CA). The abs for intracellular staining included anti-IFN-γ PE-CF594 (clone XMG1.2), anti-TNF-α PE-Cy7 (clone MP6-XT22), anti-IL-2 Brilliant Violet 421 (clone JES6-5H4), anti-CTLA-4 PE (clone UC10-4F10-11) (BD Biosciences), anti-perforin allophycocyanin (clone eBioOMAK-D) and anti-granzyme B PE (clone NGZB) (eBiosciences, San Diego, CA). A LIVE/DEAD Fixable Aqua Dead Cell Stain Kit (Invitrogen Molecular Probes, Eugene, OR) was used for dead cell exclusion. All conjugated Abs were titrated as previously reported [22].

A total of 1 x 10^6^ splenocytes/ml of medium were cultured with S*Tc*A (1 μg/ml) for 12 h at 37°C and 5% CO_2_. During the last 11 h of culture, Brefeldin A (1 μg/ml) and monensin (0.7 μg/ml) (BD Pharmingen) were present. The splenocytes were adjusted to 1 x 10^6^ cells/tube and labeled with LIVE/DEAD Fixable Aqua for 20 min in darkness at room temperature. To determine the T cell function, the cells were subsequently stained with anti-CD3, anti-CD8 and anti-CD4 mAbs, followed by fixation and permeation for intracellular staining with anti-IFN-γ, anti-TNF-α, anti-IL-2, anti-perforin and anti-granzyme B for 30 min at 4°C. To determine the frequency of the T cells that expressed the inhibitory receptors, the cells were stained with the anti-CD3, anti-CD8, anti-CD4, anti-PD-1, anti-2B4, and anti-CD160 mAbs, followed by fixation and permeation for intracellular staining with anti-CTLA-4 for 30 min at 4°C. In each experiment, non-stimulated cells were included as a negative control. At least 50,000 events, gated on live CD3^+^ cells, were acquired through flow cytometry using a FACSAria III flow cytometer (BD Immunocytometry Systems, San Jose, CA), and the results were subsequently analyzed using FlowJo 9.3.2 software (Tree Star, Ashland, OR). The gates for positivity in the multicolor panels were determined using fluorescence-minus-one control staining as recommended [23]. The analyses of the co-expression of the inhibitory receptors and polyfunctional responses were performed using a Boolean gating strategy. The data were analyzed and visualized using Pestle version 1.7 and SPICE version 5.3 software (the National Institutes of Health, Bethesda, MD) [24].

### Statistical analysis

The Mann–Whitney *U* test was used to evaluate the differences between two groups. The differences were considered statistically significant when *p* < 0.05. GraphPad Prism version 6.0 for Mac OS X statistics software (GraphPad Software, San Diego, CA) was used for the statistical analyses. Co-expression pie charts were compared using 10,000 permutations calculated with the SPICE software.

## Results

### Inhibitory receptor expression in CD4^+^ and CD8^+^ T cells from non-pregnant or pregnant healthy and infected mice

**Fig 1:**
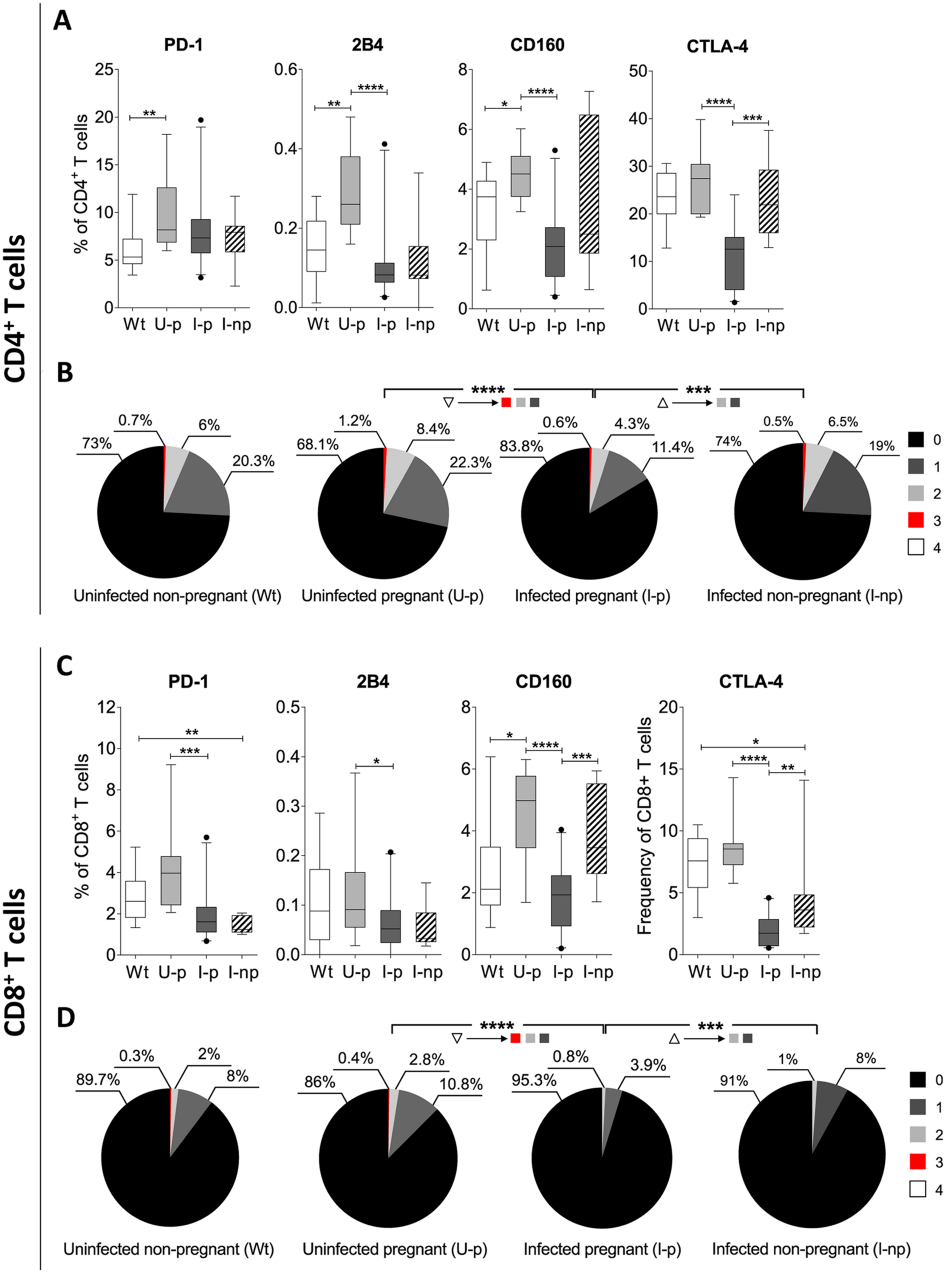
Inhibitory receptor expression in the CD4^+^ and CD8^+^ T cells from non-pregnant or pregnant uninfected mice and non-pregnant or pregnant *T*. *cruzi* infected mice. The frequencies of the CD4^+^ (A) and CD8^+^ (C) T cells that expressed PD-1, 2B4, CD160, or CTLA-4 in non-pregnant (Wt) (white box) or pregnant (U-p) (light grey box) uninfected mice and in infected pregnant (I-p) (dark grey box) or infected non-pregnant (I-np) (striped box) mice are shown. The lower and upper limits of the boxes indicate the 25th and 75th percentiles, respectively. The lines within the boxes depict the medians, and the whiskers indicate the lower and upper adjacent values (5th and 95th, respectively). The co-expression of PD-1, 2B4, CD160, and CTLA-4 in the CD4^+^ (B) and CD8^+^ (D) T cells from non-pregnant or pregnant uninfected mice and pregnant or non-pregnant *T*. *cruzi*-infected mice is shown. Increasing or decreasing frequencies of CD4^+^ (B) and CD8^+^ (D) T cells that co-express inhibitory receptors are indicated with the upward or downward arrowheads, respectively. The colors in the pie charts depict the co-expression levels of the inhibitory receptors: one (grey), two (light grey) and three (red) inhibitory receptors. The *p* values were calculated using the Mann–Whitney *U* test (A and C). The *p* values of the permutation test in the co-expression analysis (B and D) are shown in the pie charts (*p<0.05, **p<0.01, ***p<0.001, ****p<0.0001).

To determine the effect of gestation on the regulation of adaptive immune-cell function, we evaluated the expression of inhibitory receptors (PD-1, 2B4, CD160, and CTLA-4) on the CD4^+^ and CD8^+^ T cells from non-pregnant and pregnant BALB/c naive mice. The results showed that the pregnant mice had significantly higher percentages of CD4^+^ T cells that expressed PD-1, 2B4 or CD160 and CD8^+^ T cells that expressed CD160 than those observed in the non-pregnant mice (Fig 1A and 1C) (*p*<0.01 or *p*<0.05).

With the aim of determining whether the immune regulation mechanism that prevents rejection of the fetus in naive mice was modified in the context of chronic infection by *T*. *cruzi*, the frequencies of the CD4^+^ and CD8^+^ T cells that expressed or co-expressed the inhibitory receptors in the uninfected and infected pregnant mice were compared. Significantly lower frequencies of CD4^+^ T cells that expressed 2B4, CD160 or CTLA-4 were observed in the group of *T*. *cruzi*-infected pregnant mice than in the uninfected (healthy) pregnant mice (Fig 1A) (p<0.0001). Among the infected mice, a statistically significant increase in the frequency of CD4^+^ T cells expressing CTLA-4 was observed in infected non-pregnant regarding that of the infected pregnant mice (Fig 1A) (p<0.001). The evaluation of the co-expression of PD-1, 2B4, CD160, and CTLA-4 showed that significantly lower frequencies of CD4^+^ T cells that co-expressed two or three inhibitory receptors were found in *T*. *cruzi*-infected pregnant mice than in the uninfected pregnant mice (Fig 1B) (*p*<0.0001). Among the infected mice, a statistically significantly increase in the frequency of CD4^+^ T cells that co-expressed two inhibitory receptors was observed in infected non-pregnant mice compared to the infected pregnant mice (Fig 1B) (*p*<0.001). Furthermore, evaluation of the expression of the inhibitory receptors in the CD8^+^ T cells showed significantly lower frequencies of CD8^+^ cells that expressed PD-1, 2B4, CD160 or CTLA-4 in the infected pregnant mice than in the uninfected pregnant mice (Fig 1C) (*p*<0.001 or *p*<0.0001). Such significant decrease in the frequency of CD8^+^ T cells expressing CD160 or CTLA-4 was significantly reversed in infected non-pregnant mice (Fig 1C) in regards to the infected pregnant mice (*p*<0.001 or *p*<0.0001). Finally, evaluation of the co-expression of these inhibitory receptors showed a significantly lower frequency of CD8^+^ T cells that co-expressed two or three molecules in the infected pregnant mice group than in the uninfected pregnant mice (Fig 1D) (*p*<0.0001), whereas in infected non-pregnant mice there was a significant increase in the frequency of CD8^+^ T cells that co-expressed two and those that expressed one inhibitory receptor with regard to the infected pregnant mice group (Fig 1D) (*p*<0.001). Taken together, these results indicate that the regulation of the maternal immune system induced during gestation can be modulated by external factors such as *T*. *cruzi* infection.

### Polyfunctional profiles of T cells from pregnant and non-pregnant infected mice

**Fig 2:**
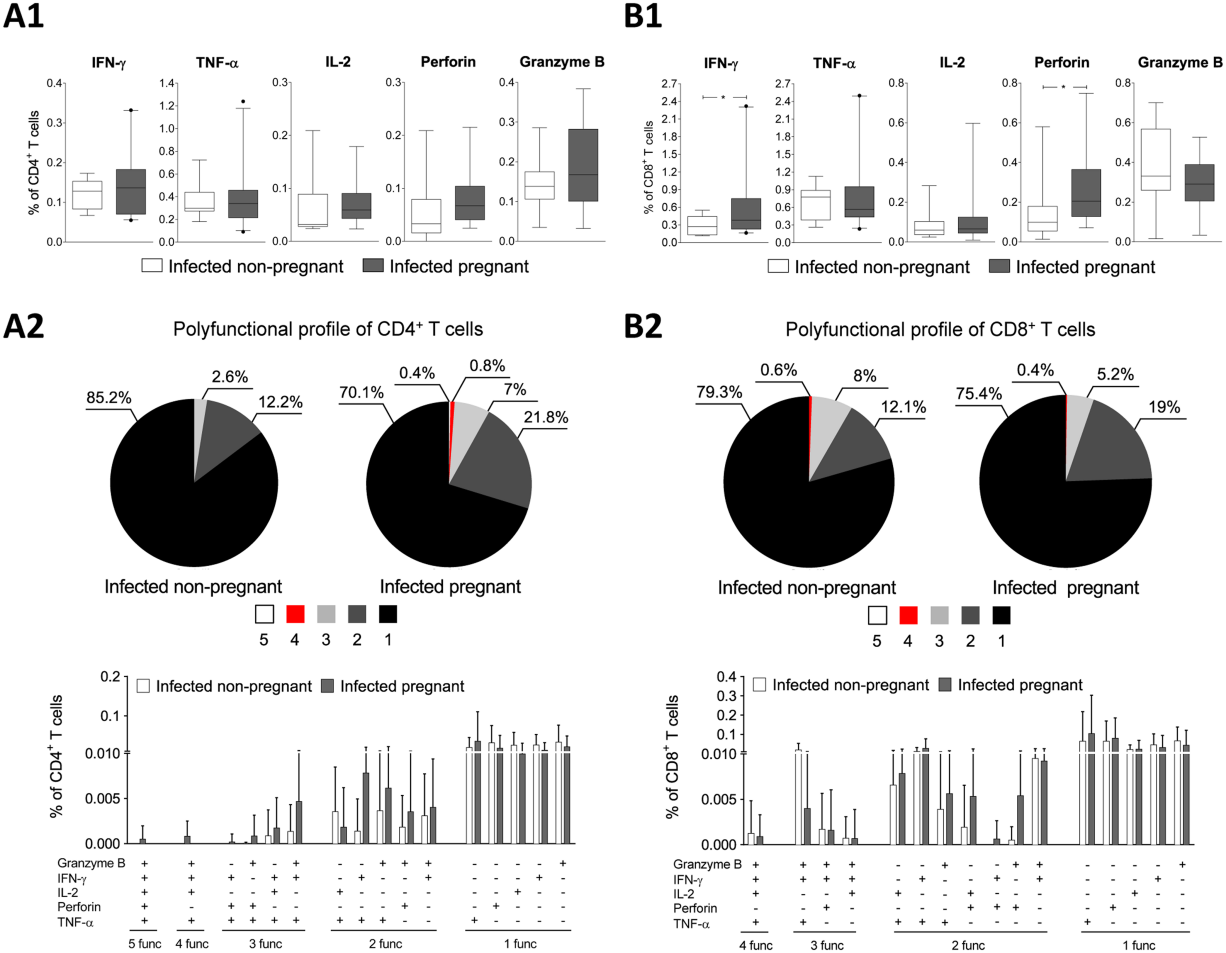
Polyfunctional profiles of the CD4^+^ and CD8^+^ T cells from infected mice. The frequency of the CD4^+^ (A1) and CD8^+^ (B1) T cells that expressed IFN-γ, TNF-α, IL-2, perforin and granzyme B in infected non-pregnant (white box) and pregnant mice (grey box) is shown. Panels A2 and B2 show the percentages of polyfunctional activity of the T cells from infected pregnant and non-pregnant mice, determined by the simultaneous measurements of the granzyme B, IFN-γ, IL-2, perforin and TNF-α expression after S*Tc*A stimulation. Panels A2 and B2 also show the frequency of the 16 most prevalent combinations of these functional molecules in CD4^+^ (A2) and CD8^+^ T cells (B2) from infected non-pregnant (white box) and pregnant mice (grey box) after stimulation with S*Tc*A. The functional profiles are grouped and color-coded according to the number of functions, as shown in the pie charts. A positive cytokine response was defined as a frequency of 0.05%, determined as the average frequency of the CD4^+^ or CD8^+^ T cell response obtained from non-infected mice after stimulation with S*Tc*A following subtraction of the values for cells cultured without antigen.

To determine whether the observed patterns of expression and co-expression of the inhibitory receptors was associated with the T cell functions, we evaluated the responses of the *T*. *cruzi*-specific CD4^+^ and CD8^+^ T cells from the pregnant and non-pregnant mice with chronic *T*. *cruzi* infections. Specifically, the production of the intracellular cytokines (IL-2, IFN-γ and TNF-α) and cytotoxic molecules (granzyme B and perforin) after *Tc*SA stimulation was determined. The obtained results showed that in the infected pregnant mice compared to infected non-pregnant mice there was not a significantly higher percentage of CD4^+^ T cells expressing IL-2^+^, perforin^+^ and granzyme B^+^ (Fig 2A1) but there was a statistically significant higher percentage of CD8^+^ T cells expressing IFN-γ^+^ and perforin^+^ (Fig 2B1) (*p*<0.05). In order to evaluate the polyfunctional response the frequency of the 16 most prevalent combinations of these functional markers in the *T*. *cruzi*-specific CD4^+^ and CD8^+^ T cells from the infected non-pregnant and pregnant mice are shown in Fig 2A2 and 2B2. The results showed that in the infected pregnant mice, the frequency of polyfunctional CD4^+^ T cells tended to be greater than in the non-pregnant mice although these differences were not statistically significant. In fact, CD4^+^ T cells that demonstrated five (granzyme B^+^, IFN-γ^+^, IL-2^+^, perforin^+^, TNF-α^+^) or four (granzyme B^+^, IFN-γ^+^, IL-2^+^, TNF-α^+^) functions were detected only in the pregnant mice (Fig 2A2). Similarly, a 4% increase in the frequency of the CD4^+^ T cells that expressed three markers (mainly granzyme B^+^, IFN-γ^+^, TNF-α^+^) and a10% increase in the frequency of CD4^+^ T cells that expressed two markers (mainly IFN-γ^+^, TNF-α^+^) were observed in the pregnant mice compared with the non-pregnant mice (Fig 2A2). Regarding the frequency of the polyfunctional CD8^+^ T cells, and although these differences were not statistically significant, a 3% decrease in the percentage of CD8^+^ T cells that expressed three markers (granzyme B^+^, IFN-γ^+^, TNF-α^+^) was observed in the pregnant mice compared with the non-pregnant mice. However, a 7% increase in the frequency of CD8^+^ T cells that expressed two markers (mainly granzyme B^+^, perforin^+^) was observed in the pregnant group of mice compared with the non-pregnant mice (Fig 2B2).

### Parasite detection in peripheral blood of infected pregnant mice and in fetal tissue

To evaluate the possible changes in the parasite load induced by the gestation-associated homeostasis, the presence of parasite DNA was evaluated in peripheral blood from mothers, before and after pregnancy. The presence of the parasite DNA was detected in 5 out of 22 infected mice before pregnancy, whereas after pregnancy, it was detected in 18 out of 22 mice (Table 1). However, within the group of the 5 mice in which the PCR was positive before pregnancy, only three of the mice were positive by PCR after becoming pregnant. Moreover, the presence of the parasite DNA after pregnancy was detected in 15 out of the 17 mice for which the PCR result before pregnancy was negative. The evaluation of the parasites in the fetal tissue showed that the *T*. *cruzi* DNA was detected in 7 out of 18 fetuses from mothers that demonstrated positive PCR results after pregnancy (Table 1). Conversely, in mothers with negative PCR results after pregnancy, congenital transmission was not detected. In addition, it was found that the parasitemia levels were moderately high and similar between the mice that transmitted the parasite (ct mean = 27.7) and the mice that did not transmit it (ct mean = 27.6) (S1 Table).

**Table 1:** *T*. *cruzi* detection in peripheral blood of pregnant mice and fetuses by conventional polymerase chain reaction.

*T*. *cruzi* detection by polymerase chain reaction			No. of mice that transmit *T*. *cruzi* to fetuses (%)
(*n* = 22)			
Before pregnancy	After pregnancy		
*T*. *cruzi* detection	*T*. *cruzi* detection	No. of samples (%)	
Negative (-)	+	15 (88.2%)	6 (40%)
(*n* = 17)	-	2 (11.8%)	0 (0%)
Positive* (+)	+	3 (60%)	1 (33%)
(*n* = 5)	-	2 (40%)	0 (0%)

*The result was considered positive when at least one PCR (kinetoplast or satellite) was positive (detection of 350 bp and 195 bp fragments, respectively, resolved by electrophoresis on 2% agarose gels containing ethidium bromide). Each set of reactions included a control negative (genomic DNA from a non-infected mice), and positive (genomic DNA of *T*. *cruzi*, 1 ng).

### Patterns of inhibitory receptor expression and polyfunctional profiles of T cells associated with congenital transmission from infected pregnant mice

**Fig 3:**
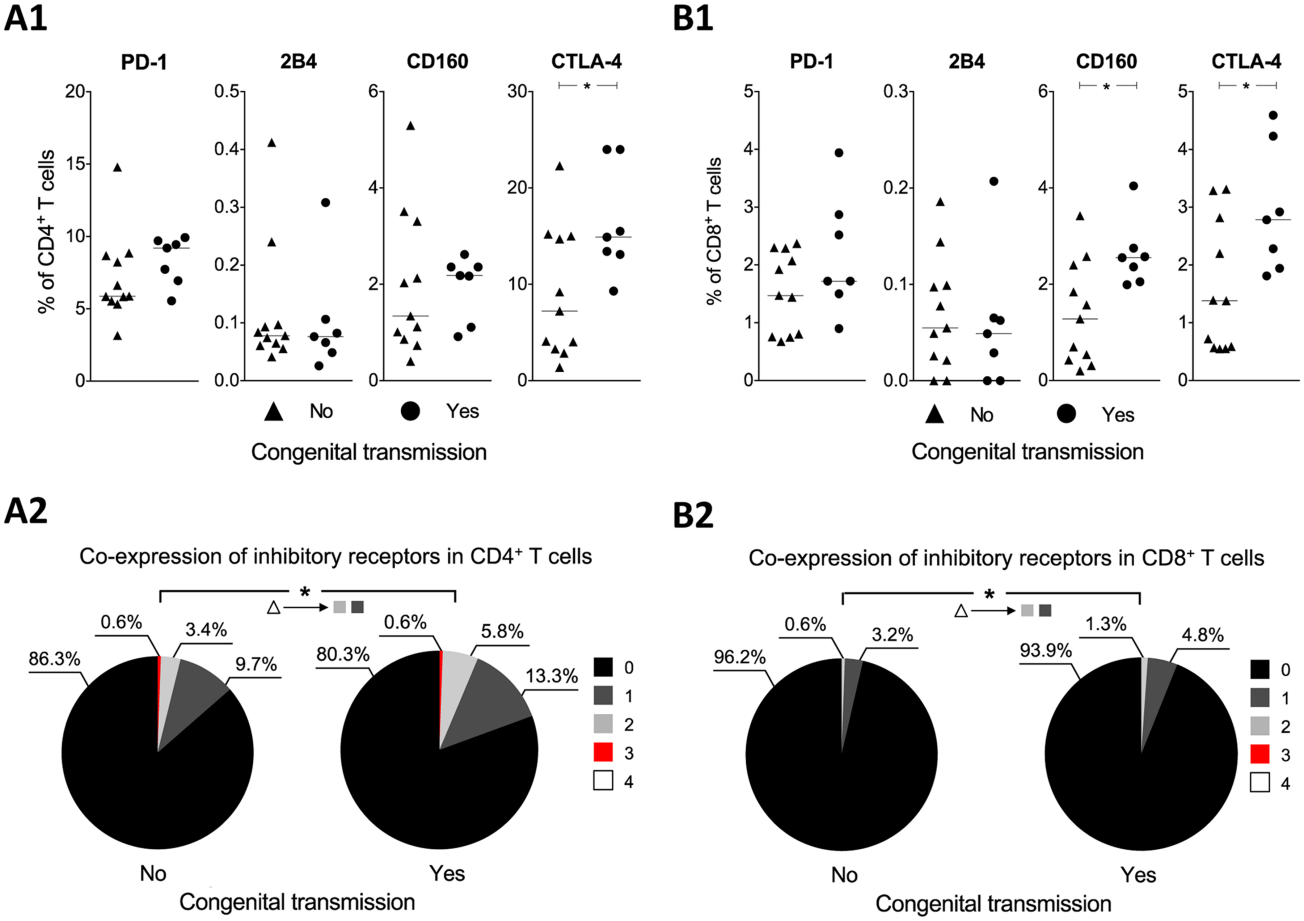
Inhibitory receptor expression from CD4^+^ and CD8^+^ T cells in infected pregnant mice with detectable parasitemia by PCR. The frequencies of the T cells that expressed PD-1, 2B4, CD160, or CTLA-4 from mothers that did not transmit (black triangle) or did transmit the parasite (black circle) (A1 and B1) are shown. The co-expression of the inhibitory receptors in T cells from mothers that did not transmit and did transmit the parasite is shown. The colors in the pie charts indicate the co-expression of inhibitory receptors (A2 and B2). Increasing and decreasing frequencies of inhibitory receptor co-expression are indicated, respectively, with upward and downward arrowheads and with color-coded according to the number of inhibitory receptors that co-express. The *p* values were calculated using the Mann–Whitney *U* test (A1 and B1). The *p* values of the permutation test in the co-expression analysis (A2 and B2) are shown in the pie charts (*p<0.05).

**Fig 4:**
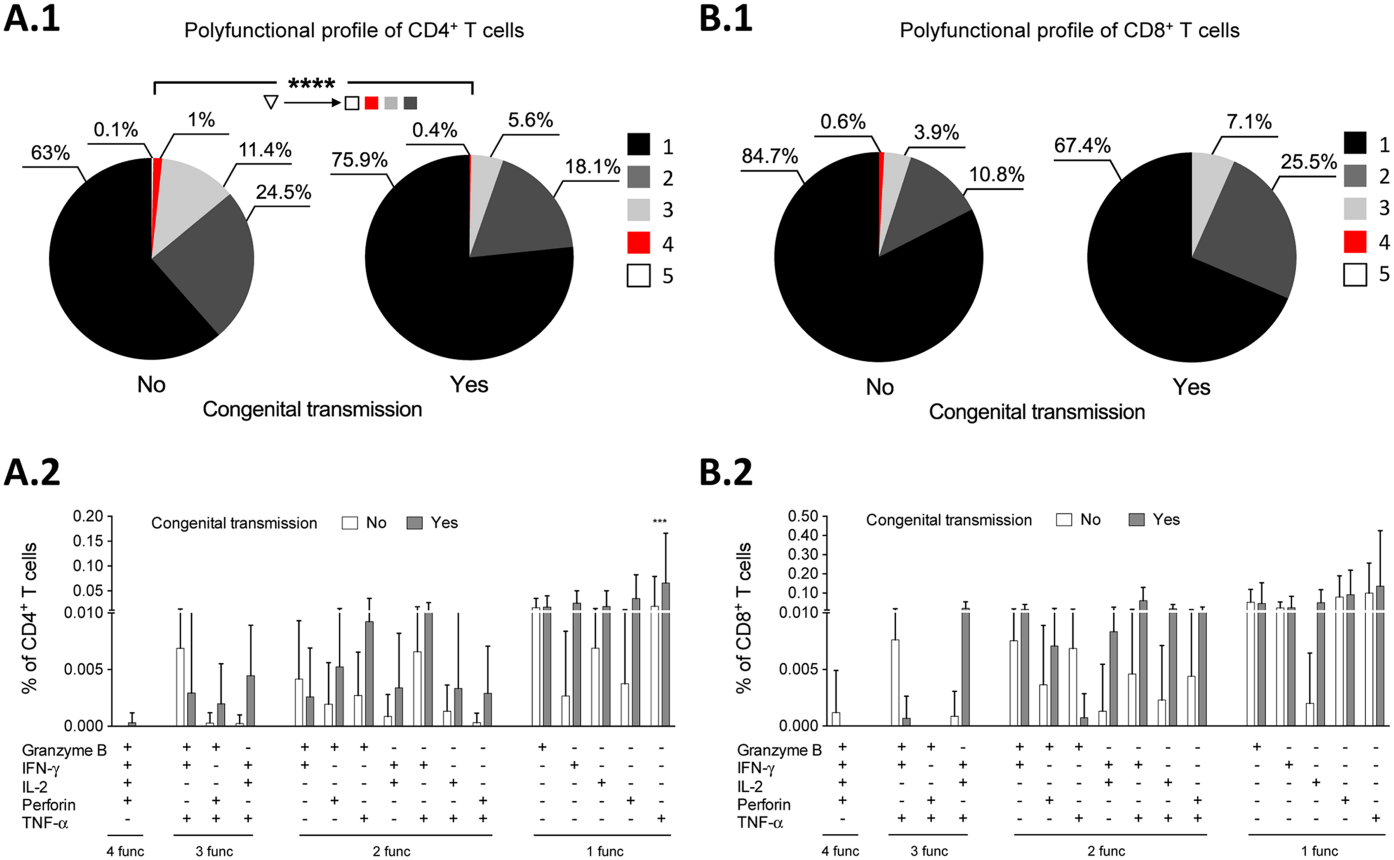
Polyfunctional profile of the *T*. *cruzi*- CD4^+^ and CD8^+^ T cells response from infected pregnant mice with detectable parasitemia by PCR. The functional activity of CD4^+^ (A1) and CD8^+^ (B1) T cells from non-transmitting and transmitting mice, determined using a five-function assay to simultaneously measure the expression of granzyme B, IFN-γ, IL-2, perforin and TNF-α after stimulation with S*Tc*A is shown. The frequency of the parasite-specific CD4^+^ (A2) and C8^+^ (B2) T cells that produced 16 distinct combinations of five functions after stimulation with S*Tc*A in non-transmitting (white bars) and transmitting mice (grey bars) is shown. The functional profiles are grouped and color-coded according to the number of functions, as shown in the pie charts. Increasing or decreasing frequencies of polyfunctional T cells are indicated with the upward or downward arrowheads, respectively. The *p* values of the permutation test in the co-expression analysis (A1 and B1) are shown in the pie charts. The *p* values in A2 and B2 panels were calculated using the Mann–Whitney *U* test (***p<0.001, ****p<0.0001).

On the basis that gestation modulated the inhibitory receptor expression and the polyfunctionality of the CD4^+^ and CD8^+^ T cells in *T*. *cruzi*-infected mice, the inhibitory receptor expression and polyfunctionality profiles of T cells were evaluated in infected pregnant mice that transmitted or did not transmit the parasite to their progeny. A significantly higher frequency of CD4^+^ cells that expressed CTLA-4 was observed in the group of mothers that transmitted the parasite than in the mothers that did not transmit it *p*<0.05 (Fig 3A1). When the co-expression of inhibitory receptors was evaluated, significantly higher frequencies of CD4^+^ T cells that co-expressed two and those that expressed one inhibitory receptor were observed in the mice that transmitted the parasite *p*<0.05 (Fig 3A2). When the expression of individual inhibitory receptors was evaluated in the CD8^+^ T cells, significantly higher frequencies of CD8^+^ T cells that expressed CD160 or CTLA-4 were found in the group of mothers that transmitted *T*. *cruzi* compared to the mothers that did not transmit it *p*<0.05 (Fig 3B1). Similarly, evaluation of the co-expression of inhibitory receptors showed that a significantly higher frequency of CD8^+^ T cells that co-expressed two and those that expressed one molecule were present in the group of transmitting mice *p*<0.05 (Fig 3B2). On the basis that the quality of the T cells response is critical for the control of chronic infections such as Chagas disease, we also evaluated the polyfunctional profiles of the CD4^+^ and CD8^+^ T cells following stimulation with S*Tc*A. A decrease in the frequency of polyfunctional CD4^+^ (*p*<0.0001) and CD8^+^ T cells (Fig 4A1 and 4B1, respectively) and an increase in the frequency of monofunctional CD8^+^ T cells were observed in the group of transmitting mothers. The frequency of the 16 most prevalent combinations of functional markers in the parasite-specific CD4^+^ and CD8^+^ T cells from the infected pregnant mice with positive PCR results are shown in Fig 4B1 and 4B2. In the group of transmitting mice, the most prevalent population was the monofunctional CD4^+^ T cells producing TNF-α (*p*<0.001) (Fig 4B1). Furthermore, the CD8^+^ T cells that exhibited four functions (granzyme B^+^, IFN-γ^+^, IL-2^+^, perforin^+^) were only detected in the mothers who did not transmit the parasite although it had no statistically significance (Fig 4B2). Likewise, the mothers that did not transmit the parasite showed a higher frequency of CD8^+^ T cells that produced granzyme B, IFN-γ, and TNF-α compared with the mothers that transmitted the parasite (Fig 4B2).

## Discussion

To prevent rejection of the fetus, a transient suppression of maternal cell-mediated immunity is induced during gestation. This suppression is characterized by decreased effector functions of CD8^+^ T cells and a lower frequency of CD4^+^ T cells [25]. Thus, in pregnancy, the immune system has the duties of defending the mother and the fetus from foreign pathogens while simultaneously tolerating the paternal alloantigens expressed by the fetus [26]. The regulation of the immune effector functions is mediated by cellular and molecular interactions with numerous cellular components of the immune system and also by a vast amount of molecules with immunoregulatory capacities, such as inhibitory receptors [10]. However, although these immune mechanisms are needed for fetal protection, they can be a risk factor for the congenital transmission of infectious diseases. In this manner, the immunoregulation during gestation may be involved in mother-to-child transmission of *T*. *cruzi*, which has become important in endemic and non-endemic countries. Thus, the aim of this study was to determine the immunological modifications induced by gestation in an experimental model of chronic *T*. *cruzi* infection with regard to the expression of the inhibitory receptors that modulate T-cell functions and influence this immunoregulation of parasite congenital transmission. In spite of the DTU of the infecting parasite is most often unknown together to the fact that mixed infections have been described, herein two *T*. *cruzi* strains (DTUI and DTU V) were used for the experimental chronic infection in order to evaluate the influence of the parasite genetic variability in the mechanisms of immunoregulation during pregnancy and the parasite congenital transmission. However, given that no differences in the pattern of functional response and inhibitory receptor expression were observed between mice infected with DA or SOL strains (S1 Fig), the obtained results were shown together. In spite of the human monocytes and whole blood cells isolated from healthy donor which were *in vitro* infected with culture-derived trypomastigotes showed slight differences in the monocyte activation and cytokine production depending on the infecting strain [27], a similar expression pattern of cytokines and cytotoxic molecules was observed by the parasite-specific CD8^+^ and CD4^+^ T cells from mice infected with SOL and DA strains following stimulation with total antigens from different *T*. *cruzi* strains (S2 Fig). Consequently, in the present manuscript solely *T*. *cruzi* soluble antigens (S*Tc*A) from Y strain were used for *in vitro* stimulation.

Several immunomodulatory mechanisms appear to be crucial in regulating the maternal immune cell responses during pregnancy, including molecules that generate negative signals that regulate the activation of CD4^+^ and CD8^+^ T cells [28]. In particular, PD-1 has been described as playing an important role in maternal-fetal tolerance by inducing apoptosis of paternal antigen-specific T cells during pregnancy [29]. In fact, PD-1 ligand blockage *in vivo* resulted in increased abortion rates and reduced litter sizes [30]. Previous studies have also shown the involvement of CTLA-4 in the suppression of activated T cells [31]. Nevertheless, during pregnancy, CTLA-4 blockage did not interfere with the protective effect of the Treg cells [32]. Others molecules including CD160 and 2B4, which regulate cytotoxic activity and cytokine production [3], might play critical roles in the delicate balance between effective immunity and maternal tolerance to the fetal allograft. Similarly, our results showed that during gestation in uninfected (healthy) mice, an up-regulation of inhibitory receptors was induced, perhaps as a mechanism for the maintenance of maternal-fetal tolerance. Interestingly, although the frequency of the CD4^+^ and CD8^+^ T cells that expressed inhibitory receptors tended to increase during gestation in healthy mice, the percentage of CD4^+^ and CD8^+^ T cells that co-expressed these molecules was similar in pregnant and non-pregnant healthy mice. This finding suggests that this up-regulation was a physiological mechanism induced by the pregnancy but was not a sign of T cell exhaustion.

In this study, gestation in a murine model was found to induce a differential immunological pattern depending on the status of the mice (healthy or *T*. *cruzi* infected). Specifically, in healthy mice, gestation led to significant increases in the individual expression of the inhibitory receptors on the CD4^+^ and CD8^+^ T cells, perhaps as a mechanism involved in the feto-maternal tolerance. However, in mice chronically infected with *T*. *cruzi*, gestation induced a significant decrease in the frequency of T cells that expressed or co-expressed inhibitory receptors, an increase in the frequency of polyfunctional CD4^+^ and CD8^+^ T cells and an increase in the parasite load. This different immunological profile induced by the infection during pregnancy may be due to the breakdown of the gestation-induced immune homeostasis, probably to control the parasite load. Interestingly, this pattern of reversing the process of immune homeostasis and increased polyfunctionality in CD4^+^ and CD8^+^ cells appeared to be directly related with no *T*. *cruzi* congenital transmission. Thus, our results show that the mice that transmitted the parasite have an increase in the frequency of both the CD4^+^ and CD8^+^ cells that co-expressed the inhibitory receptors and a decrease in the polyfunctionality of these T cells. These results suggest that females who are not able to reverse the biological profile induced by pregnancy are those that transmit the parasite, which demonstrates the importance of the balance in the immune response to keep the fetus alive and to prevent the parasite transmission.

Consistent with previous reports [12, 14, 33], our results showed that there was a detectable increase in the parasitemia level (measured as a positive conventional PCR results after gestation) during gestation in the *T*. *cruzi*-infected mice and that those mice who transmitted the infection had a positive PCR during gestation. However, when the parasitemia was assessed by quantitative PCR in mice with a positive PCR result (transmitting and non-transmitting the infection) similar levels of parasitemia were detected in all the mice, those that transmitted and those who did not transmit the infection. These data indicate that although the parasitemia in pregnant mice seems to be an important factor that contributes to the congenital transmission of the parasite, it is not a determining factor. Thus, our data indicate that an effective response of the maternal T cells is critical for reducing the chances of parasite transmission from the mother to the fetus. In agreement with this, other authors have suggested that monocytes from parasite-transmitting women are less activated than those from non-transmitting women [34]. In fact, chronically infected non-pregnant women have increased levels of circulating TNF-α. In women that do not transmit the parasite, these levels remain elevated during gestation. In contrast, pregnant women that transmit the parasite showed a down-regulation in the secretion of the IFN-γ and TNF-α [35, 36]. Together, and based on the fact that there were no differences in the parasitemia levels among mice that transmitted and those who did not transmit the infection, these results show that the quality and/or capacity of the T cell response can be considered as a determining factor in the congenital transmission of *T*. *cruzi*. This is consistent with the ecological model of multiple and complex interactions among parasites, mothers, placentae and fetuses [7]. Thus, the results shown in this manuscript suggest that the immunological profile of a mother during pregnancy could be an useful tool for determining the risk factor of vertical transmission of the *T*. *cruzi* parasite. Evaluation of these profiles would facilitate the subsequent clinical follow-up of the mothers and the children.

## Supporting information

S1 Table Serological detection of anti-*T*. *cruzi* IgG antibodies and parasite detection in peripheral blood of pregnant mice. (DOCX)

S1 Fig Functional profiles and inhibitory receptor expression in CD4^+^ and CD8^+^ T cells from non-pregnant and pregnant infected mice. Frequency of the T cells that expressing one of the cytokines (IFN-γ), one of the cytotoxic molecules (perforin), and one of the inhibitory receptors (PD-1) evaluated in this study, in pregnant (I-p) and non- pregnant (I-np) mice infected with SOL (black triangle) (four mice) or DA strain (black hexagon) (three mice).
(TIF)

S2 Fig Polyfunctional profiles of the CD4^+^ and CD8^+^ T cells from infected non-pregnant mice. Frequency of CD4^+^ (A1 and C1) and CD8^+^ (A2 and C2) T cells expressing IFN-γ, TNF-α, IL-2, perforin and granzyme B after stimulation with *T*. *cruzi* total antigens (strains DA (white bars), SOL (light grey bars), and Y (dark grey bars)), in non-pregnant mice infected with DA (A) or SOL (C) strains. Polyfunctional activity of CD4^+^ (B1 and D1) and CD8^+^ (B2 and D2) T cells, determined by the simultaneous measurement of the granzyme B, IFN-γ, IL-2, perforin and TNF-α is shown, in non-pregnant mice infected with DA (B) or SOL (D) strains. The functional profiles are grouped and color-coded according to the number of functions, as shown in the pie charts. The *p* values were calculated using the Mann–Whitney *U* test (A and C). The *p* values of the permutation test in the co-expression analysis (B and D) are shown in the pie charts (*p<0.05, ***p<0.001, ****p<0.0001).
(TIF)
