# Supplementary material for: Expression of inhibitory receptors and polyfunctional responses of T cells are linked to the risk of congenital transmission of T. cruzi
Source: PLoS Negl Trop Dis. 2017 Jun 9;11(6):e0005627. doi: 10.1371/journal.pntd.0005627 (PMC5479596; doi:10.1371/journal.pntd.0005627)
Supplement: S1 Table — (DOCX) [file pntd.0005627.s001.docx]

|  | **Code** | **Parasite strain** | **IgG antibodies^a^ (O.D)** | **Conventional PCR** | | **qPCR^b^**  **ct** | **Parasite transmission** |
| --- | --- | --- | --- | --- | --- | --- | --- |
|  |  |  |  | **Before Pregnancy** | **After Pregnancy** |  |  |
|  | 1-3 | DA | 0.28 | **+** | **+** | **25.2** | **+** |
|  | 1-4 | DA | 0.40 | **+** | **+** | 29.4 | - |
|  | 2-2 | DA | 0.82 | - | **+** | 31.1 | - |
|  | 2-3 | DA | 0.39 | **+** | - | 34.8 | - |
|  | 3-1 | DA | 0.17 | - | **+** | 31.2 | - |
|  | 3-3 | DA | 0.24 | - | **+** | **30.0** | **+** |
|  | 3-5 | DA | 0.21 | - | **+** | **32.5** | **+** |
|  | 3-6 | DA | 0.21 | - | **+** | 33.4 | - |
|  | 4-2 | DA | 0.56 | - | - | 35.2 | - |
|  | 4-4 | DA | 0.41 | - | **+** | 25.1 | - |
|  | 5-2 | DA | 0.48 | - | **+** | **24.7** | **+** |
|  | 5-4 | DA | 0.27 | - | - | 34.3 | - |
|  | 6-3 | DA | 0.30 | - | **+** | **29.8** | **+** |
|  | 8-1 | SOL | 0.98 | - | **+** | 25.3 | - |
|  | 8-6 | SOL | 0.63 | **+** | **+** | 23.6 | - |
|  | 9-1 | SOL | 0.47 | **+** | - | 34.5 | - |
|  | 9-3 | SOL | 0.60 | - | **+** | 26.6 | - |
|  | 10-4 | SOL | 0.44 | - | **+** | **26.9** | **+** |
|  | 11-4 | SOL | 0.78 | - | **+** | 29.7 | - |
|  | 12-3 | SOL | 0.20 | - | **+** | 23.3 | - |
|  | 13-1 | SOL | 0.23 | - | **+** | **25.1** | **+** |
|  | 13-3 | SOL | 0.13 | - | **+** | 25.4 | - |

^a^ The anti-parasite IgG levels were determined using an ELISA directed against the *T. cruzi* soluble antigens (*Tc*SA) as previously described [14]. Whole blood was collected into tubes without anticoagulant and incubated at 37ºC for 30 min. The samples were refrigerated for 30 min at 4ºC and centrifuged at 13,000 rpm for 10 min.

^b^ Each PCR reaction contained 200 ng genomic DNA, 0.5 μM T. cruzi satellite repeat DNA-specific primers, 10 μl Eva Green Master Mix (BioRad), and sterile H_2_O to a final total volume of 20 μl. Separately, reactions containing 200 ng genomic DNA, 0.5 μM of β-actin F 5’ AGAGGGAAATCGTGCGTGAC-3’ and β-actin R 5′-CAATAGTGATGACCTGGCCGT-3′ primers, 10 μl of Eva Green Master Mix (BioRad) and sterile H_2_O to a final total volume of 20 μl were used as loading controls. Non-parasite DNA (ct): (34.2). Standard curve for *T. cruzi* DA strain (ct): 1 ng(12.0), 0.1(15.4), 0.01(19.2), 0.001(23.1), 0.0001(26.2). Standard curve for *T. cruzi* SOL strain (ct): 1 ng(9.4), 0.1(12.7), 0.01(17.0), 0.001(21.4), 0.0001(24.3).
