# Supplementary figures and images for: Expression of inhibitory receptors and polyfunctional responses of T cells are linked to the risk of congenital transmission of T. cruzi
Source: PLoS Negl Trop Dis. 2017 Jun 9;11(6):e0005627. doi: 10.1371/journal.pntd.0005627 (PMC5479596; doi:10.1371/journal.pntd.0005627)

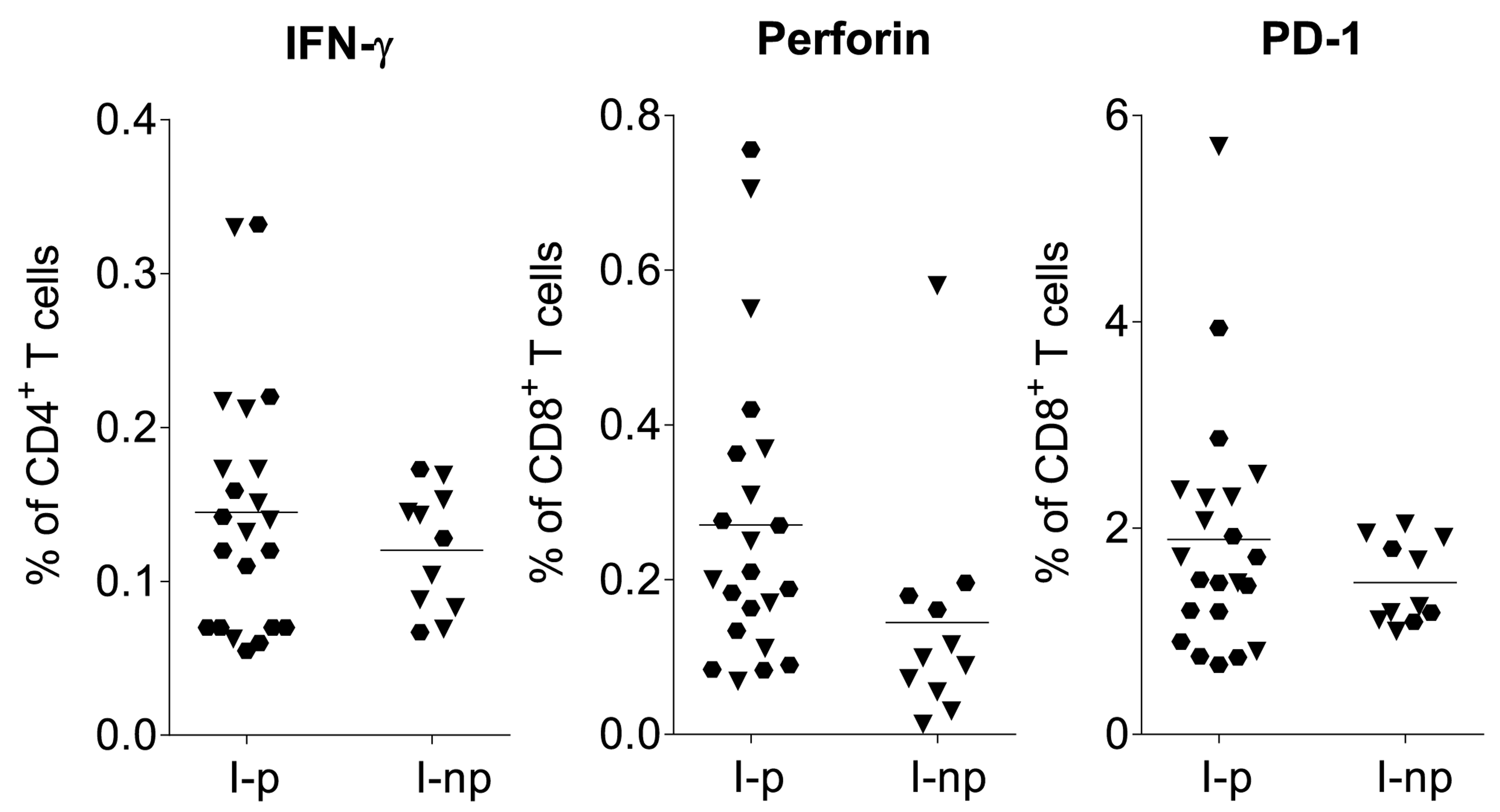

Supplement: S1 Fig — Frequency of the T cells that expressing one of the cytokines (IFN-γ), one of the cytotoxic molecules (perforin), and one of the inhibitory receptors (PD-1) evaluated in this study, in pregnant (I-p) and non- pregnant (I-np) mice infected with SOL (black triangle) (four mice) or DA strain (black hexagon) (three mice). (TIF) [file pntd.0005627.s002.tif]

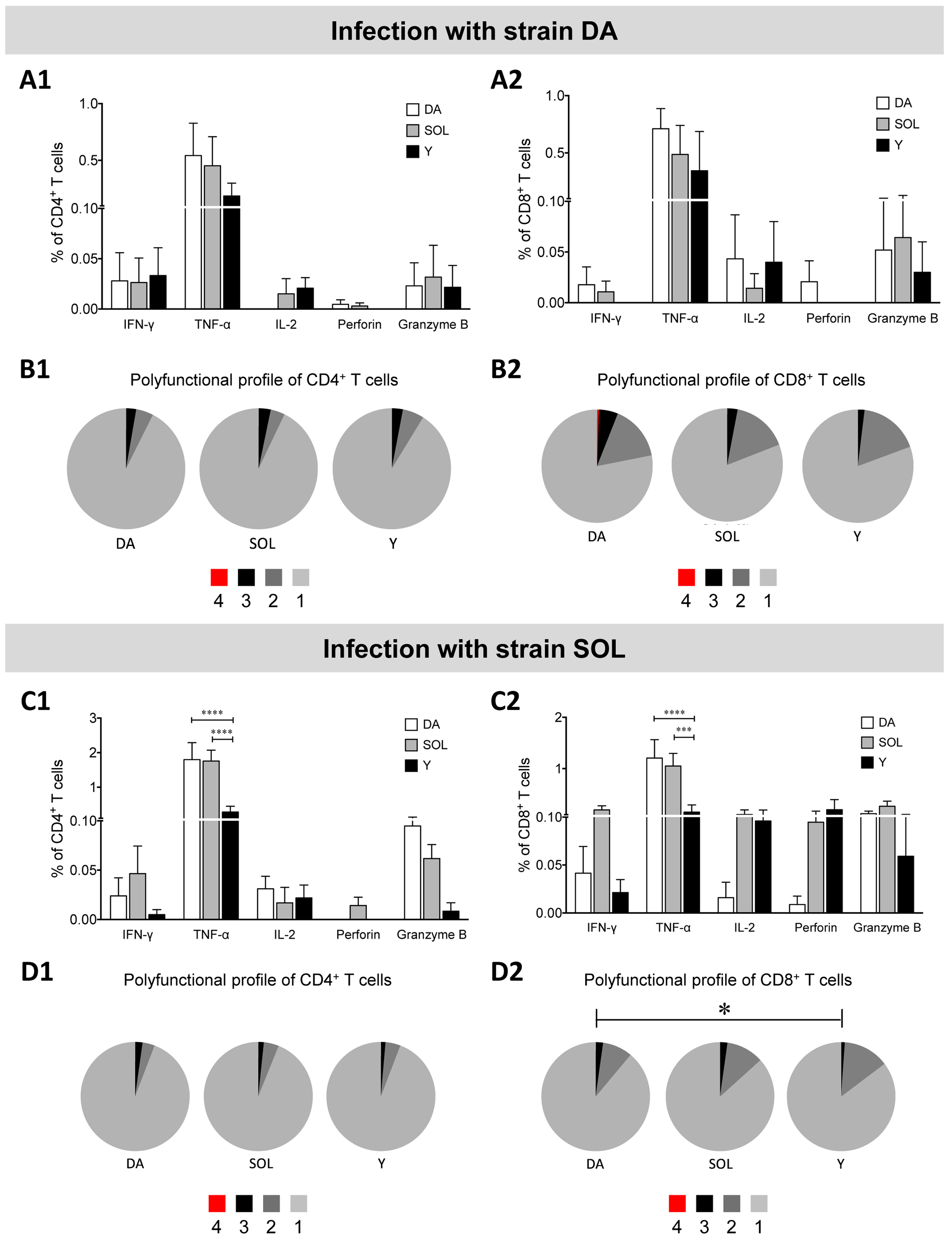

Supplement: S2 Fig — Frequency of CD4+ (A1 and C1) and CD8+ (A2 and C2) T cells expressing IFN-γ, TNF-α, IL-2, perforin and granzyme B after stimulation with T. cruzi total antigens (strains DA (white bars), SOL (light grey bars), and Y (dark grey bars)), in non-pregnant mice infected with DA (A) or SOL (C) strains. Polyfunctional activity of CD4+ (B1 and D1) and CD8+ (B2 and D2) T cells, determined by the simultaneous measurement of the granzyme B, IFN-γ, IL-2, perforin and TNF-α is shown, in non-pregnant mice infected with DA (B) or SOL (D) strains. The functional profiles are grouped and color-coded according to the number of functions, as shown in the pie charts. The p values were calculated using the Mann–Whitney U test (A and C). The p values of the permutation test in the co-expression analysis (B and D) are shown in the pie charts (*p<0.05, ***p<0.001, ****p<0.0001). (TIF) [file pntd.0005627.s003.tif]
